# Commonly Used Pancreatic Stellate Cell Cultures Differ Phenotypically and in Their Interactions with Pancreatic Cancer Cells

**DOI:** 10.3390/cells8010023

**Published:** 2019-01-05

**Authors:** Daniela Lenggenhager, Manoj Amrutkar, Petra Sántha, Monica Aasrum, Johannes-Matthias Löhr, Ivar P. Gladhaug, Caroline S. Verbeke

**Affiliations:** 1 Department of Pathology, Institute of Clinical Medicine, University of Oslo, Blindern, 0316 Oslo, Norway; Daniela.Lenggenhager@usz.ch (D.L.); c.s.verbeke@medisin.uio.no (C.S.V.); 2 Department of Pharmacology, Institute of Clinical Medicine, University of Oslo, Blindern, 0316 Oslo, Norway; monica.aasrum@medisin.uio.no; 3 Department of Pathology and Molecular Pathology, University Hospital Zürich, University of Zürich, Schmelzbergstrasse 12, 8091 Zürich, Switzerland; 4 Department of Hepato-Pancreato-Biliary Surgery, Institute of Clinical Medicine, University of Oslo, P.O. Box 1171 Blindern, 0318 Oslo, Norway; i.p.gladhaug@medisin.uio.no; 5 Department of Pathology, Oslo University Hospital Rikshospitalet, Nydalen, 0424 Oslo, Norway; petra.santha@medisin.uio.no; 6 Department of Clinical Science, Intervention and Technology, Karolinska Institutet, K 53, 141 86 Stockholm, Sweden; matthias.lohr@ki.se; 7 Department of Hepato-Pancreato-Biliary Surgery, Oslo University Hospital Rikshospitalet, Nydalen, 0424 Oslo, Norway

**Keywords:** pancreatic stellate cells, pancreatic cancer, tumor-stroma interaction, TGF-β

## Abstract

Activated pancreatic stellate cells (PSCs) play a central role in the tumor stroma of pancreatic ductal adenocarcinoma (PDAC). Given the limited availability of patient-derived PSCs from PDAC, immortalized PSC cell lines of murine and human origin have been established; however, it is not elucidated whether differences in species, organ disease status, donor age, and immortalization alter the PSC phenotype and behavior compared to that of patient-derived primary PSC cultures. Therefore, a panel of commonly used PSC cultures was examined for important phenotypical and functional features: three primary cultures from human PDAC, one primary from normal human pancreas, and three immortalized (one from human, two from murine pancreas). Growth rate was considerably lower in primary PSCs from human PDAC. Basal collagen synthesis varied between the PSC cultures, and TGF-β stimulation increased collagen synthesis only in non-immortalized cultures. Differences in secretome composition were observed along with a divergence in the DNA synthesis, migration, and response to gemcitabine of PDAC cell lines that were grown in conditioned medium from the various PSC cultures. The findings reveal considerable differences in features and functions that are key to PSCs and in the interactions with PDAC. These observations may be relevant to researchers when selecting the most appropriate PSC culture for their experiments.

## 1. Introduction

Pancreatic ductal adenocarcinoma (PDAC) has a poor prognosis with an overall 5-year survival of less than 7% [1]. Because survival has only minimally improved in the past decades, PDAC is predicted to become the second leading cause for cancer-related mortality by 2030 [2,3]. The two main reasons for poor patient outcome are late diagnosis and pronounced resistance to most conventional treatment options [4]. The tumor stroma, which is exceedingly prominent in PDAC, plays an important role in treatment resistance [5], although the exact mechanisms by which it contributes to drug resistance are currently poorly understood. Whereas in the past, the stroma was mainly considered a mere mechanical barrier to drug delivery [6], it is now increasingly attributed a more active, multifaceted role, in which pancreatic stellate cells (PSCs) take center stage by exerting effects on the cancer cells through paracrine mechanisms as well as the production of various components of the extracellular matrix (ECM) [7,8,9,10]. Approaches to target the stroma therapeutically have currently been undertaken with conflicting results. While inhibition of the hedgehog pathway led to enhanced efficacy of gemcitabine in a mouse model [11], a clinical study with the same approach failed to show any benefit in patient outcome [12]. Moreover, two recent mouse studies demonstrated that stromal depletion either by inhibition of the hedgehog pathway or genetic depletion of myofibroblasts resulted in increased tumor aggressiveness and reduced survival [13,14]. 

From these apparently inconsistent results, the concept of dual, both tumor-promoting and tumor-restraining, effects of the stroma emerged [15], highlighting the need for more in-depth preclinical research in order to exploit the stroma therapeutically in a more predictable manner. Given the central role of the PSCs in the production, maintenance, and remodeling of the ECM as well as in the complex, reciprocal interactions with the cancer cell population, multiple PSC culture model systems have been developed [16]. Whereas initially, murine PSCs were mainly employed for in vitro experiments, the body of literature on studies with human PSCs has steadily grown during the past 10 years [17]. However, as the availability of primary patient-derived PSCs from PDAC is limited, isolation is time consuming, culturing hampered by considerable variability between individual preparations, and passaging limited due to senescence, several immortalized PSC cell lines of both human and murine origin have been established, usually by the transfection with SV40 large T antigen and human telomerase (hTERT) [18,19,20]. Only four years ago, a PSC culture from normal human pancreas (HPaSteC) has become commercially available [21]. 

Despite obvious differences in species, origin from normal or diseased pancreas, and the primary or immortalized nature of the PSC cultures, results from studies using various PSC cultures have been published and discussed without due consideration of the potential impact of these differences on the study findings [22]. Indeed, a systematic characterization of the various PSC cultures that would underpin the assumed comparability of data is lacking. The aim of this study was therefore to characterize a panel of different PSC cultures that are commonly used to study human PDAC, including primary PSCs –PDAC-derived PSCs and commercially available PSCs from normal human pancreas (HPaSteC), as well as immortalized PSCs that were isolated from human pancreas with chronic pancreatitis (RLT-PSC) [18] and immortalized PSCs of murine origin (i-mPSC clone 2 and clone 3) [20]. Key phenotypical and functional PSC features such as morphology, protein expression, proliferation rate, collagen synthesis, response to transforming growth factor-beta (TGF-β), secretome composition, and the effect of soluble factors produced by PSCs on DNA synthesis, migration activity, and gemcitabine sensitivity of pancreatic cancer cells were analyzed. The findings of the study reveal marked differences for some of the above features, which may help researchers in selecting the most appropriate PSC culture for their experiments.

## 2. Materials and Methods

### 2.1. Patients

The study protocol and patient consent documents were approved by the Regional Committee for Medical and Health Research Ethics (REC South East, project number 2015/738) and followed the Helsinki Declaration. Written informed consent was obtained from the three adult patients, whose tumor tissue was used for the study. 

### 2.2. Reagents

Reagents were purchased from the following sources: BODIPY™ 493/503, Dulbecco’s modified Eagle’s medium (DMEM) containing 4.5 g/L glucose, DMEM F-12 containing GlutaMAX-I, penicillin-streptomycin (Pen-Strep), amphotericin B, trypsin/EDTA, fetal bovine serum (FBS), and Pierce^TM^ BCA protein assay kit from Thermo Fisher Scientific (Waltham, MA, USA); bovine serum albumin (BSA), dimethyl sulfoxide (DMSO), gemcitabine hydrochloride, 3-(4,5-Dimethylthiazol-2-yl)-2,5-Diphenyltetrazolium Bromide (MTT), phosphate buffered saline (PBS), and senescence cells histochemical staining kit from Sigma–Aldrich (St. Louis, MO, USA); TGF-β from R&D Systems Europe (Abingdon, UK); human anti-alpha smooth muscle actin (α-SMA; BS66) from Nordic Biosite AB (Taby, Sweden); anti-glial fibrillary acidic protein (GFAP) (GA5), anti-epithelial cell adhesion molecule (EpCAM; VU1D9), anti-vimentin (D21H3), anti-GAPDH and Smad2/3 antibody sampler kit from Cell Signaling Technology (Beverly, MA, USA); anti- TGF-β receptor I (TGF-β RI) and anti-TGF-β receptor II (TGF-β RII) from Abcam (Cambridge, UK); secondary HRP-conjugated antibodies goat anti-mouse and goat anti-rabbit IgG from Bio-Rad Laboratories (Hercules, CA, USA); secondary Alexa Fluor-conjugated antibodies (anti-mouse and anti-rabbit) and DAPI from Jackson ImmunoResearch (West Grove, PA, USA).

### 2.3. Isolation and Culture

Primary human pancreatic stellate cells (hPSCs) were obtained from tumor tissue sampled from surgical resection specimens with PDAC. Human pancreatic stellate cells were isolated and cultured by the outgrowth method as previously described [23,24]. Cultures were established and propagated from three different patients (designated hPSC-1, hPSC-2, and hPSC-3). The purity of the hPSCs was assessed by morphology and demonstration of α-SMA and vimentin expression. All experiments were performed using cell cultures between passage 3 and 8. The PDAC cell lines BxPC-3 and MIA PaCa-2 were purchased from ATCC (Manassas, VA, USA). The hPSC and PDAC cell lines were cultured and maintained in DMEM supplemented with 10% FBS, 1% Pen-Strep, and 1% amphotericin B. HPaSteC (Human Pancreatic Stellate Cells, fibroblastic cells isolated from pancreas of a 22-week old, fetal, non-diseased, male donor) were purchased from ScienCell Research Laboratories (San Diego, CA, USA), cultured, and maintained according to the supplier’s protocol.

Immortalized PSC cell lines from human RLT-PSC (referred to as i-hPSC) [18] and from mice (referred to as i-mPSC C2 and i-mPSC C3) [20] were kindly provided by Prof. J-M. Löhr, Karolinska Institute, Sweden. The immortalized cells were cultured and maintained in DMEM/F-12 supplemented with 10% FBS, 1% Pen-Strep and 1% amphotericin B.

### 2.4. Morphology, H&E Staining, and Size Measurement

Cells were cultured in 96-well plates, fixed in 4% formaldehyde, and stained with hematoxylin and eosin (H&E). Images were captured under a light microscope. The cell shape was assessed in each PSC culture, and the length, width, and area of 50 cells were measured using FIJI software (National Institutes of Health, Bethesda, MA, USA).

### 2.5. Immunocytochemistry

Cells were cultured in 96-well plates, fixed in 4% formaldehyde, blocked in 5% BSA in PBS, and incubated overnight with anti-α-SMA (1:50) and anti-vimentin (1:200). Positive cells were visualized by secondary antibodies Alexa Fluor 488 (anti-mouse) and Alexa Fluor 594 (anti-rabbit) conjugated secondary antibodies (1:200), and DAPI was used for nuclear staining. For the detection of lipid droplets, cells were stained using BODIPY and counterstained with DAPI. Images were captured using EVOS FLoid Cell Imaging Station (Thermo Fisher Scientific). The percentage of positive cells was counted in ten randomly selected fields (20× magnification) from each culture. 

### 2.6. Western Blot Analysis

Cells were washed with PBS, and total cell lysates were prepared by boiling for 5 min in Laemmli buffer (4% SDS, 20% glycerol, and 120 mM Tris-HCl, pH 6.8) with the addition of 2% bromophenol blue and 5% β-mercaptoethanol. Aliquots of protein were separated on 10% polyacrylamide gels by electrophoresis (SDS-PAGE). The proteins were transferred to nitro-cellulose membranes using a semidry transfer system (Bio-Rad). The membranes were blocked in Tris-buffered saline containing 0.1% Tween 20 (TBST) with 5% non-fat dry milk solution and incubated with the primary antibodies as indicated (in TBST with 5% non-fat dry milk or BSA) overnight at 4 °C. The blots were then washed 3 times in TBST and incubated with HRP-conjugated secondary antibodies at room temperature for 1 h. The blots were visualized with LumiGLO^®^ (KPL, Gaithersburg, MD, USA). Densitrometic analysis of the immunoblots was obtained with Labworks Software (UVP, Cambridge, UK). 

###  2.7. Cell Proliferation

#### 2.7.1. Growth Curves

Growth curves and doubling times were determined by counting the number of viable cells derived from freshly trypsinized cells in triplicates. Approximately 5000 cells/well were seeded in 24-well plates, and the cell number was counted every 24 h for 5 days. Cells were cultured and maintained according to the culture maintenance protocol for the respective PSC cultures. The doubling time of the cell population was calculated from the logarithmic growth curve using the following formula:

$$Doubling\;Time\;\left(h\right)=\frac{duration\;log\left(2\right)}{log\left(Number\;of\;cells\;at\;last\;counting\right)-log\left(Number\;of\;cells\;at\;first\;counting\right)}$$

#### 2.7.2. MTT-Based Proliferation Assay

Approximately 3000 cells/well were seeded in 96-well plates, and the change in the number of viable cells over a period of 48 h was calculated using the MTT assay, according to manufacturer’s instructions. Briefly, at 24 and 72 h after seeding, the cell viability was determined spectrophotometrically at 570 nm by measuring the formation of purple-colored formazan crystals. The proliferation rate was calculated as the percentage change in the number of viable cells relative to the time interval.

### 2.8. Collagen Synthesis

Collagen synthesis was measured by quantification of [^3^H]-proline incorporation into acetic acid-soluble proteins, as described previously [23,25]. Briefly, cells were seeded in 24-well plates at a density of 20,000 cells/cm^2^ and cultured overnight. The following day, the medium was replaced with serum-free DMEM (SFM) and incubated for an additional 24 h. Subsequently, the medium was replaced with fresh SFM containing 100 μg/mL ascorbic acid, 100 μg/mL 3-aminopropionitrile, and 2 μCi/mL [^3^H]-proline. The reaction was stopped after 24 h by addition of 50 μL/mL 10 N acetic acid. The cells were lysed by adding 100 μL 0.2 N acetic acid per well, and protein concentration was determined spectrophotometrically at 595 nm using Bradford protein assay. The incorporation of [^3^H]-proline into collagen was determined by liquid scintillation counting. The panel of various PSC cultures were incubated with TGF-β (10 nM) for 24 h prior to incubation with the proline mix to determine the stimulatory effect on collagen synthesis. 

### 2.9. Preparation of Conditioned Media

To obtain PSC-conditioned medium (PSC-CM), sub-confluent PSC cultures in 10-cm^2^ Petri dishes were washed thoroughly with PBS and incubated with fresh SFM (~10 mL) for 48 h. This conditioned medium from the cultures was collected, centrifuged, and stored at 20 °C until further use. 

### 2.10. Effect of PSC-CM on Pancreatic Cancer Cells

#### 2.10.1. DNA Synthesis

The effect of PSC-CM on DNA synthesis in PDAC cells was measured by the thymidine incorporation assay, as previously described [23]. Briefly, BxPC-3 and MIA PaCa-2 cells were seeded in 12-well plates at a density of 10,000 cells/cm^2^, and cultured overnight in DMEM. The medium was replaced with SFM and incubated for additional 24 h. The cells were then stimulated with PSC-CM for 24 h and pulsed with [^3^H]-thymidine (1 μCi/mL) for the last 4 h of stimulation. The incorporation of [^3^H]-thymidine was determined by liquid scintillation counting. 

#### 2.10.2. Migration Assay

The effect of PSC-CM on cancer cell migration was evaluated using the scratch assay, as described previously [26]. Briefly, confluent BxPC-3 and MIA PaCa-2 cells seeded in 12-well plates were cultured with SFM overnight. Next day, scratch wounds were made, followed by incubation with SFM or PSC-CM for 24 h in respective wells. The scratch wounds were observed in an Olympus inverted microscope with a 4× objective. Pre-marks under the bottom of the wells consisting of one horizontal and several vertical ink lines per well served to ensure that images were taken at the same observation field for time point 0 h and 24 h. Images taken immediately after the addition of SFM (control group) and PSC-CM (0 h) and at 24 h were obtained with an Olympus F-View II Soft Imaging System High-Resolution CCD Camera. For each picture, the wound area was measured by FIJI software, as previously described [27]. Percent wound closure was calculated for the time point of observation based on the mean of eight observations from each PSC-CM relative to the wound closure in SFM.

#### 2.10.3. Chemosensitivity

BxPC-3 and MIA PaCa-2 cells were cultured in 96-well plates at a density of 3000 cells/well and treated for 48 h with gemcitabine at a final concentration range of 0.01 μM to 100 μM. The cell viability and IC_50_ values were determined using the MTT assay, in which the degree of formazan crystals formation is relative to the number of viable cells. IC_50_ is the amount of gemcitabine required for inhibition of cancer cell growth by 50% compared to untreated controls. Furthermore, cancer cells were also incubated with PSC-CMs from the PSC panel for 24 h prior to treatment with gemcitabine for 48 h at a final concentration of 10 μM. The response to gemcitabine was evaluated with the MTT assay. 

### 2.11. Secretome Analysis

The PSC-CM samples (~10 mL) were concentrated down to 450 µL using 10 kDa cut off Amicon Ultra centrifugal filter devices. Subsequently, the proteins were reduced, alkylated, and in-solution digested with trypsin (Promega) overnight at 37 °C. The resulting peptides were desalted and concentrated before mass spectrometry (MS) by the STAGE-TIP method using a C18 resin disk (3M Empore). Each peptide mixture was analyzed in three technical replicates by nEASY-LC coupled to QExactive Plus (ThermoElectron, Bremen, Germany) with EASY Spray PepMap^®^ RSLC column (C18, 2 µm, 100Å, 75 µm × 50 cm). The resulting MS raw files were submitted to MaxQuant software version 1.6.1.0 for protein identification and label-free quantification using the Andromeda search engine. Carbamidomethyl (C) was set as a fixed modification, and protein N-acetylation and methionine oxidation were set as variable modifications. First search peptide tolerance of 20 ppm and main search error 4.5 ppm were used. Trypsin without the proline restriction enzyme option was used, with two allowed miscleavages. The minimal unique + razor peptide number was set to 1, and the allowed false discovery rate (FDR) was 0.01 (1%) for peptide and protein identification. Label-free quantification (LFQ) was employed with default settings. The SwissProt human or mouse database was used for the database searches. Known contaminants as provided by MaxQuant and identified in the samples were excluded from further analysis. Perseus software, version 1.5.6.0, was used for statistical analysis. Pathway analysis of identified proteins was performed using the Kyoto Encyclopedia of Genes and Genomes (KEGG) database [28]. Gene ontology (GO) analysis was also conducted using the functional annotation tool available through the DAVID Bioinformatics Database [29,30]. The workflow of the procedure is described in Supplementary Materials Figure S1.

### 2.12. Statistical Analysis

All values are expressed as a mean ± standard error of mean (SEM). Statistical analysis of the results was performed by a two-tailed unpaired student’s *t*-test for comparison of two treatment groups. Differences were considered significant at *p* < 0.05.

## 3. Results

### 3.1. Phenotypic Characterization of the Various PSC Cultures

**Figure 1:**
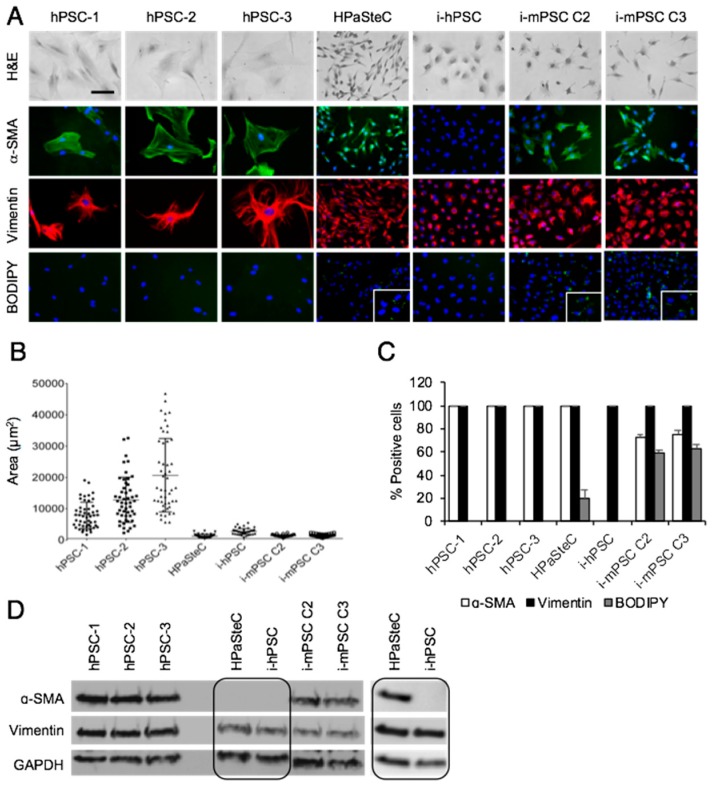
Phenotypic characterization of pancreatic stellate cells. (**A**) For morphological analysis, cells were stained with hematoxylin and eosin (H&E), BODIPY for detection of cytoplasmic lipid droplets and immunostained with anti-α-SMA (green) and anti-vimentin (red) antibodies. Nuclei were stained with DAPI (blue). Scale bar = 100 µM. (**B**) Cell size of the various PSC cultures was determined by measurement of the area of 50 cells for each PSC culture using FIJI software. (**C**) Number of positive cells for α-SMA, vimentin, and BODIPY in percentage. (**D**) Cells were lysed and proteins subjected to immunoblotting using anti-α-SMA and anti-vimentin antibodies. Due to high exposure time required for detection, α-SMA expression in HPaSteC cells is also presented in a separate blot. GAPDH was used as a loading control. PSC, pancreatic stellate cell; hPSC, human primary PDAC-derived PSC culture; HPaSteC, PSCs from normal human pancreas; i-hPSC, immortalized human PSCs; i-mPSC C2 and C3, immortalized mouse PSCs clone 2 and 3.

Hematoxylin and eosin-based morphological analysis revealed different cell morphologies in the seven PSC cultures: polygonal in hPSCs, long thin spindle-shaped in HPaSteC, small roundish in i-hPSC, and small stellate-shaped in i-mPSCs (Figure 1A). Notably, the nuclei of i-hPSC were non-spherical, mostly cleaved and often horseshoe-shaped, and as such, they differed from the spherical nuclei that were observed in the other six PSC cultures. 

Cell size distribution analysis (Figure 1B) revealed that the average cell area of hPSC was 10.9-fold and 5.1-fold higher compared to that of HPaSteC and i-hPSC, respectively, confirming the large size of hPSC, as shown in Figure 1A. Furthermore, hPSCs were heterogenous regarding cell size, whereas HPaSteC, i-hPSC, and i-mPSCs showed a relatively uniform cell size. Compared to hPSC, the length/width ratio was 1.9-fold higher and 2.0-fold lower for HPaSteC and i-hPSC, respectively, confirming the long thin cell shape of HPaSteC and the more roundish shape of i-hPSC (Figure 1A, Supplementary Materials Figure S2). As activated PSCs are known to lose their vitamin A storing cytoplasmic lipid droplets [7], BODIPY staining for the detection of neutral lipids was performed. Lipid droplets were absent in hPSCs and immortalized human PSC cultures, but present in a minority of HPaSteC cells (19.7 ± 7.8%) and to a large extent i-mPSCs (58.6 ± 2.6% and 63.0 ± 2.9% for C2 and C3, respectively; Figure 1A,C). Furthermore, expression of proteins considered characteristic of PSCs was analyzed by immunofluorescence and Western blot analysis. Independent of their origin and activation status, all PSC cultures in the panel expressed strongly the mesenchymal marker vimentin, whereas a variable expression of the PSC activation marker α-SMA was detected in six of the seven PSC cultures (Figure 1A,D). α-SMA expression was not detectable in the i-hPSC culture either by immunofluorescence or Western blot analysis (Figure 1D). Quantification of cells positive for α-SMA, vimentin, and BIODIPY is shown in Figure 1C. GFAP was not detected in any of the PSC cultures (data not shown), consistent with the reported loss of expression during culturing [31]. None of the PSC cultures showed expression of the epithelial marker EpCAM (data not shown), excluding the possibility of contamination by epithelial cells during isolation. Notably, both murine PSC cultures displayed expression of α-SMA as well as the presence of cytoplasmic lipid droplets. 

### 3.2. Growth Curves and Doubling Times

**Figure 2:**
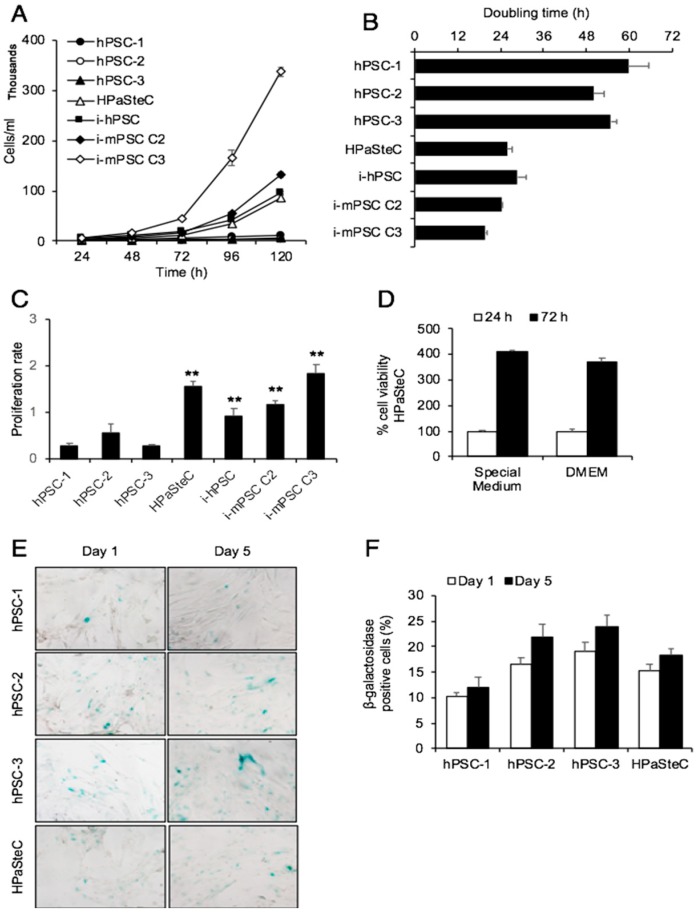
Growth curves and doubling time. Growth curves (**A**) and doubling time (**B**) were determined by counting cell numbers every 24 h for 5 days. (**C**) Cell proliferation rate was obtained by measuring the percentage of cell viability by the MTT assay at 24 and 72 h after cell seeding. (**D**) Percentage of cell viability of HPaSteC cells measured by the MTT assay at 24 and 72 h after cell seeding in special or DMEM medium. (**E**,**F**) β-galactosidase staining of primary cultures, including hPSCs and HPaSteC, performed at 24 h (day 1) and 120 h (day 5) after cell seeding (**E**) and percentage positive cells, using ImageJ software for cell counting (**F**). Data are mean ± SEM of triplicate determinations. ** *p* < 0.01 comparing average of hPSCs with HPaSteC, i-hPSC, and i-mPSCs for (**C**). PSC, pancreatic stellate cell; hPSC, human primary PDAC-derived PSC culture; HPaSteC, PSCs from normal human pancreas; i-hPSC, immortalized human PSCs; i-mPSC C2 and C3, immortalized mouse PSCs clone 2 and 3.

All PSC cultures were followed for growth (cell division) over a period of five days, and the cell numbers were counted at 24 h intervals. Immortalized cells were growing fastest and hPSCs slowest (Figure 2A). The doubling time for immortalized cells was approximately 1 day, while for hPSC it was about 2.5 days (Figure 2B). The primary PSCs from normal human pancreas (HPaSteC) were growing at a rate similar to that of i-hPSC, with a doubling time of 25.8 ± 1.4 h vs. 28.6 ± 2.6 h, respectively. These data were corroborated by a second experiment based on the MTT assay to determine the proliferation rate. Compared to the average proliferation rate of hPSCs, HPaSteC, i-hPSC, i-mPSC C2, and i-mPSC C3 showed a 4.2-, 2.4-, 3.1-, and 4.9-fold higher proliferation, respectively (Figure 2C). No significant difference in cell viability was detected between HPaSteC cells cultured in special medium or in DMEM with supplements, as used for the other primary PSC cultures (Figure 2D). β-galactosidase staining of hPSC cultures revealed that approximately 10–24% of all primary cultures, including hPSCs and HPaSteC, were positive for senescence during growth curve determination (Figure 2E,F). 

### 3.3. Collagen Synthesis

**Figure 3:**
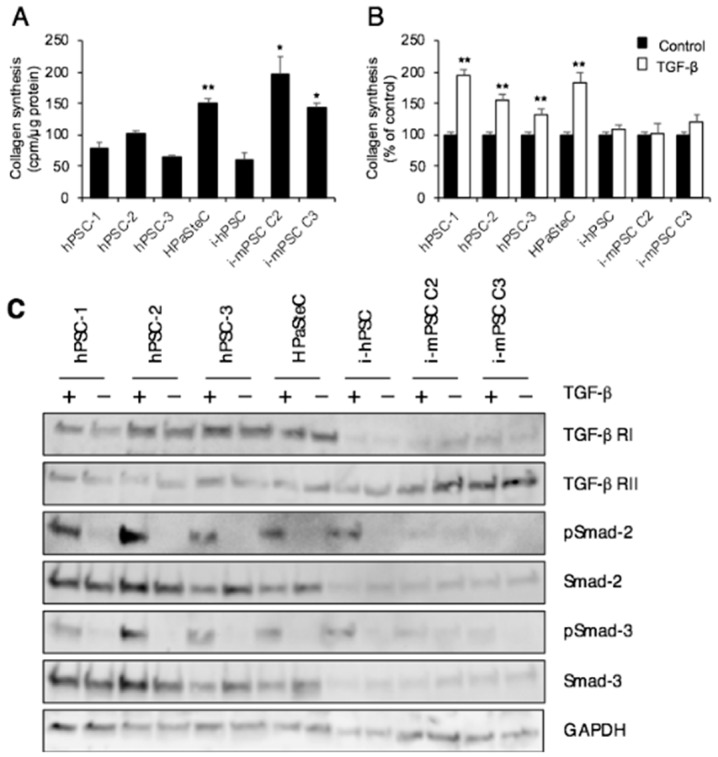
Collagen synthesis. (**A**) Basal collagen synthesis. (**B**) Collagen synthesis after TGF-β (10 nM) stimulation for 24 h. Measurement based on incorporation of [^3^H]-proline into collagen. Data are mean ± SEM of triplicate determinations. (**C**) Cells treated with or without TGF-β for 24 h were lysed and proteins subjected to immunoblotting using antibodies against TGF-β receptor I and II, phospho-Smad-2, Smad-2, phospho-Smad-3, and Smad-3. GAPDH was used as loading control. * *p* < 0.05, ** *p* < 0.01 comparing average of hPSCs with HPaSteC, i-mPSCs for (**A**); ** *p* < 0.01 comparing control (non-treated) cells with TGF-β treated cells for (**B**). PSC, pancreatic stellate cell; hPSC, human primary PDAC-derived PSC culture; HPaSteC, PSCs from normal human pancreas; i-hPSC, immortalized human PSCs; i-mPSC C2 and C3, immortalized mouse PSCs clone 2 and 3.

As activated PSCs are known to be the main producers of collagens in areas of fibrosis [32], the in vitro collagen synthesis of the seven PSC cultures was determined. Compared to hPSCs isolated from human PDAC tissue, the basal collagen synthesis was slightly lower in i-hPSC, although the difference did not reach statistical significance (Figure 3A). HPaSteC showed 1.7-fold higher basal collagen synthesis compared to the average collagen synthesis by hPSCs (Figure 3A). While cross-species comparison has its limitations, a significantly higher level of basal collagen synthesis was observed in i-mPSCs than in hPSCs (2.2- and 1.6-fold increase for cells from clone 2 and 3, respectively) (Figure 3A). Furthermore, exposure to TGF-β (10 nM) for 24 h resulted in higher collagen synthesis in all primary PSC cultures (both hPSCs and HPaSteC), while none of the immortalized PSC cultures responded significantly to TGF-β stimulation (Figure 3B). 

Protein expression analysis revealed that the immortalized PSC cultures from both human and mouse origin displayed substantially lower expression of receptor TGF-β RI compared to primary PSC cultures. No significant difference was observed in TGF-β RII expression across the panel of PSC cultures (Figure 3C). All primary PSC cultures displayed TGF-β-induced increased phosphorylation of Smad-2 and -3. Despite relatively low expression of TGF-β RI, the immortalized human PSCs displayed TGF-β-induced Smad-2, -3 phosphorylation. Phosphorylation of Smad-2, -3 was detected in neither of the immortalized mouse PSCs cultures (Figure 3C).

### 3.4. Effect of PSC-CM on Cancer Cell Proliferation, Migration, and Chemosensitivity

**Figure 4:**
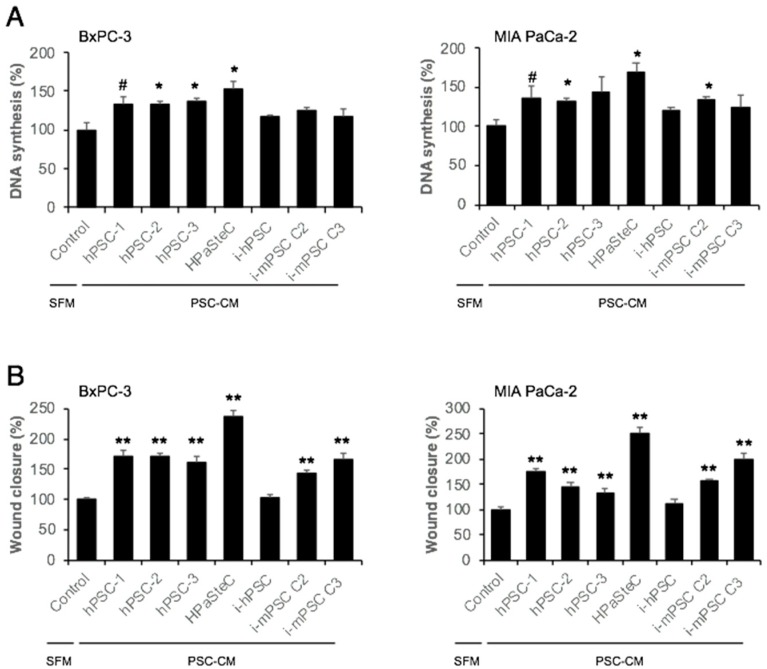
Effect of PSC-CM on pancreatic cancer cell proliferation and migration. (**A**) Cancer cell proliferation: BxPC-3 and MIA PaCa-2 cells were incubated with SFM or PSC-CMs for 24 h, and DNA synthesis was determined by [^3^H]-thymidine incorporation assay. Data are mean ± SEM of triplicate determinations. (**B**) Cancer cell migration: BxPC-3 and MIA PaCa-2 cells were cultured to confluence, and scratch wounds were established. Images of the wound area were taken immediately after the scratches and 24 h after incubation with SFM or PSC-CMs. The wound area was measured using FIJI software. Data are mean ± SEM of eight scratches for each PSC-CM. # *p* < 0.1, * *p* < 0.05, ** *p* < 0.01 comparing control (SFM) and PSC-CM. PSC, pancreatic stellate cell; hPSC, human primary PDAC-derived PSC culture; HPaSteC, PSCs from normal human pancreas; i-hPSC, immortalized human PSCs; i-mPSC C2 and C3, immortalized mouse PSCs clone 2 and 3; PSC-CM, PSC-conditioned medium; SFM, serum-free DMEM.

Next, the ability of PSC-CM to stimulate DNA synthesis and migration in BxPC-3 and MIA PaCa-2 cell lines was assessed. PSC-CM from all primary PSC cultures (both hPSCs and HPaSteC) induced DNA synthesis (fold change in the range of 1.3 to 1.7) in both cancer cell lines (Figure 4A), while PSC-CM collected from immortalized PSC cultures (both human and murine origin) showed no significant increase in DNA synthesis, except for the PSC-CM from i-mPSC C2 in MIA PaCa-2 cells. 

Migration of both BxPC-3 and MIA PaCa-2, as assessed by the scratch assay, was induced by conditioned medium from all PSC cultures, except i-hPSC (Figure 4B, Supplementary Materials Figure S3). The magnitude of migration stimulated by the PSC-CM was variable across the PSC cultures: it was highest for PSC-CM from HPaSteC (~2.5-fold in both BxPC-3 and MIA PaCa-2) and lower for PSC-CM from hPSCs and i-mPSCs (in BxPC-3 ~1.7- and ~1.5-fold, respectively; in MIA PaCa-2 ~1.5- and ~1.8-fold, respectively). 

**Figure 5:**
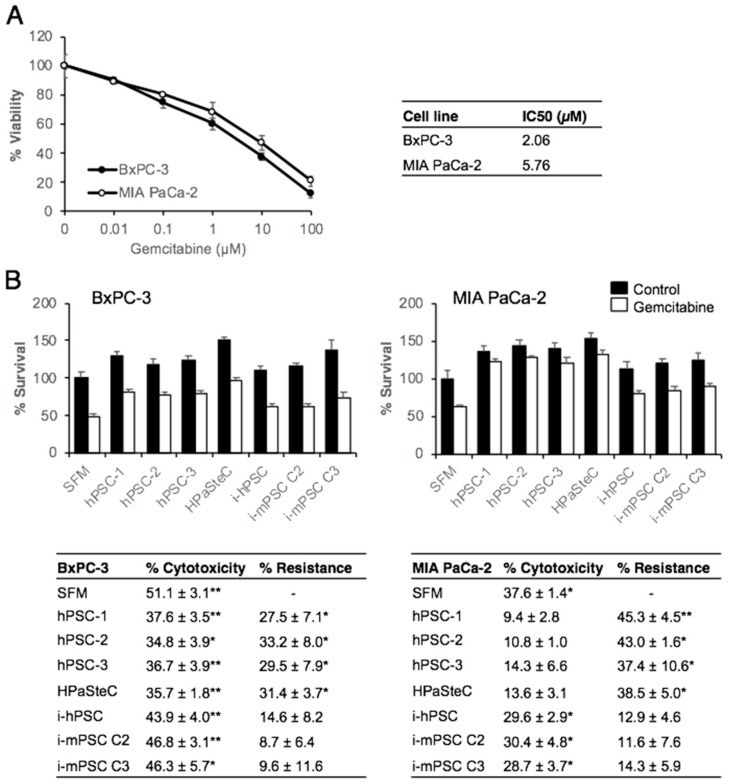
Effect of PSC-CM on chemosensitivity for gemcitabine of pancreatic cancer cells. (**A**) Gemcitabine dose response curves: BxPC-3 and MIA PaCa-2 cells (3000 cells/well) seeded on 96-well plates were incubated with varying concentrations of gemcitabine for 48 h and evaluated for cell viability using the MTT assay. IC_50_ values for gemcitabine were calculated using GraphPad Prism 4.0 software. (**B**) BxPC-3 and MIA PaCa-2 cells were incubated with PSC-CM for 24 h prior to incubation with gemcitabine (10 μM) for 48 h. Cell viability was determined using the MTT assay. Data are mean ± SEM of triplicate determinations. The table indicates gemcitabine-induced cytotoxicity in percentage and PSC-CM-induced resistance to gemcitabine, calculated by relative reduction in cytotoxicity between SFM and PSC-CM. * *p* < 0.05, ** *p* < 0.01 comparing SFM with PSC-CM. PSC, pancreatic stellate cell; hPSC, human primary PDAC-derived PSC culture; HPaSteC, PSCs from normal human pancreas; i-hPSC, immortalized human PSCs; i-mPSC C2 and C3, immortalized mouse PSCs clone 2 and 3; PSC-CM, PSC-conditioned medium; SFM, serum-free DMEM.

To evaluate the effect of PSC-CM on the chemosensitivity of BxPC-3 and MIA PaCa-2, first the dose response curves for gemcitabine in both PDAC cell lines were determined by measuring the in vitro cytotoxic effect by the MTT assay. Gemcitabine reduced cancer cell viability in a dose-dependent fashion, with a larger effect in BxPC-3 than in MIA PaCa-2 cells (Figure 5A). At a single concentration of 10 μM, the reduction in viability was 62.7 ± 2.5% and 53 ± 4.8% in BxPC-3 and MIA PaCa-2 cells, respectively (Figure 5A). The IC_50_ values suggested that BxPC-3 cells (IC_50_ = 2.7 μM) were relatively more sensitive to gemcitabine than MIA PaCa-2 cells (IC_50_ = 5.8 μM), which is in accordance with previous data [33]. It is important to note that there was a significant population in each cell line that was inherently resistant to gemcitabine, 11.9% and 20.9% in BxPC-3 and MIA PaCa-2 cells, respectively (Figure 5A). In a second step, the effect of the various PSC-CM on the chemosensitivity of BxPC-3 and MIA PaCa-2 was analyzed. Both PDAC cell lines developed significant resistance to gemcitabine following 24 h incubation with PSC-CM from the primary PSC cultures (both hPSCs and HPaSteC) (~30% for BxPC-3 and ~40% for MIA PaCa-2), while PSC-CM from all immortalized cell cultures showed no significant effect on cancer cell sensitivity to gemcitabine (Figure 5B). 

### 3.5. Secretome Analysis

**Figure 6:**
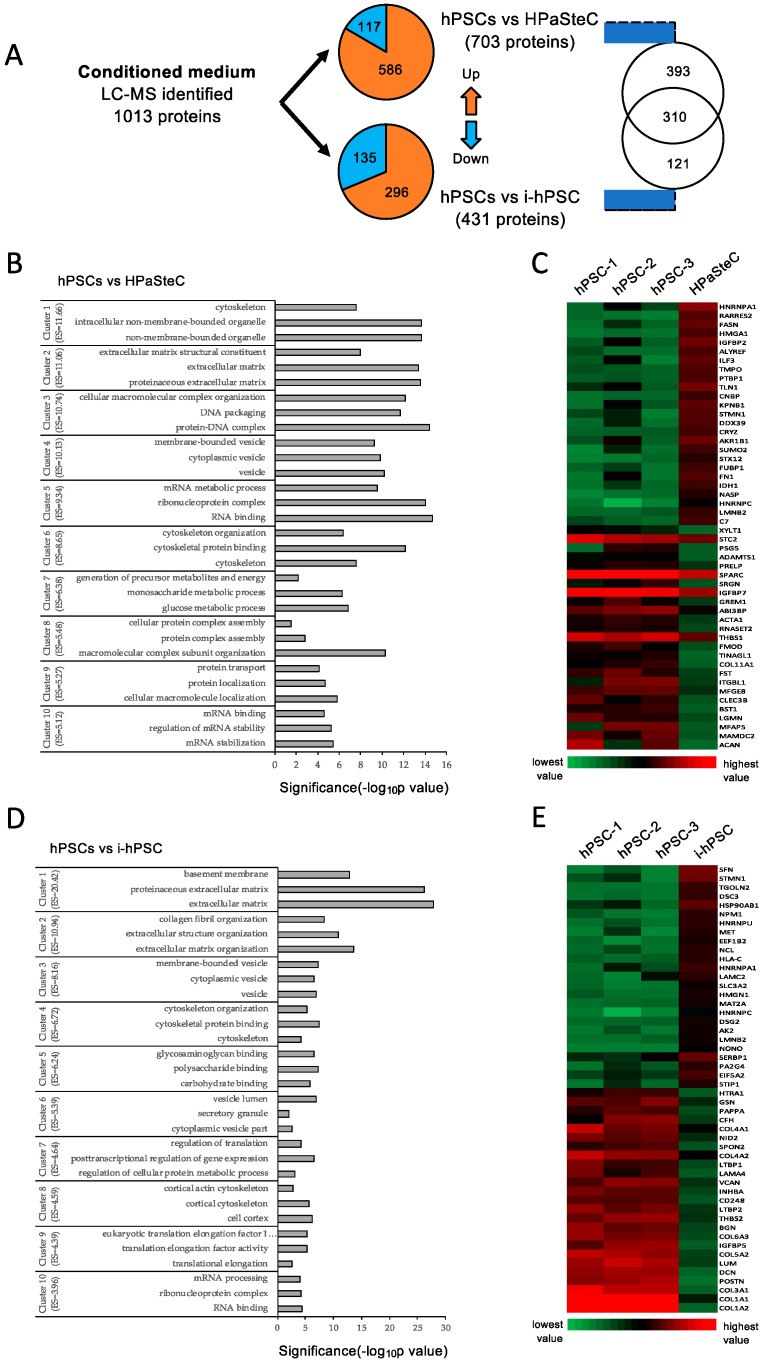
Secretome analysis of PSC-CM. Conditioned medium from various PSC cultures were subjected to proteomics analysis using LC-MS/MS. (**A**) Protein identifications from three replicates for each PSC culture and number of differentially expressed genes. (**B**,**D**) Compared to hPSC, differentially secreted proteins by HPaSteC (**B**) and by i-hPSC (**D**) detected by LC-MS were interrogated in terms of functional annotation by the DAVID Bioinformatics Resource tool. The representative GO terms cluster groups with top 10 enrichment score are presented. The horizontal axis represents the significance (*p*-value) for each term, while the vertical axis represents the GO categories. (**C**,**E**) Heatmap of protein abundance pattern for the 50 most significantly downregulated and upregulated proteins (fold change). Red and green color indicates high and low expression, respectively. GO, Gene ontology; PSC, pancreatic stellate cell; hPSC, human primary PDAC-derived PSC culture; HPaSteC, PSCs from normal human pancreas; i-hPSC, immortalized human PSCs; PSC-CM, PSC-conditioned medium.

The composition of conditioned medium from various PSC cultures was investigated by proteomics-based analysis of the secretome preparations. A total of 1013 different proteins (*Homo sapiens*) were identified in PSC-CM from hPSC, HPaSteC, and i-hPSC. A complete list of all proteins together with their identification parameters are provided in Supplementary Materials Table S1. Among these secreted proteins, 703 and 431 proteins were differentially secreted by HPaSteC and i-hPSC cells, respectively, compared to hPSCs. The majority of these differentially secreted proteins were upregulated (83% and 69% in PSC-CM from HPaSteC and i-hPSC, respectively), whereas the remainder were downregulated (Figure 6A; Supplementary Materials Table S2). 

To determine the functional role of the differentially secreted proteins, a GO analysis was undertaken using the DAVID bioinformatic resource tool, which revealed a significant representation of categories mainly related to ECM, in particular “ECM structure”, “ECM assembly”, and “collagen fibril organization” (Figure 6B,D). STRING based in-depth analysis of these differentially regulated proteins showed that the majority of them are involved in biological processes such as cellular component organization, ECM organization, ECM assembly and disassembly, as well as in other functions including RNA and protein binding. These differentially secreted proteins belong to structural cellular components such as extracellular vesicles, exosomes, and membrane bound vesicles. KEGG pathways analysis suggested that the majority of proteins belong to the following pathways: ECM-receptor interaction, focal adhesion, and metabolic pathways. A heatmap of the 50 most differentially expressed proteins showed that the major ECM components—collagens—were expressed at a significantly lower level in immortalized PSC cultures than in hPSCs and HPaSteC (Figure 6C,E, Supplementary Materials Figure S1 and Table S3). Secretome analysis of conditioned medium from mouse immortalized PSCs led to the identification of a total of 876 different proteins (*Mus musculus*) in PSC-CM from i-mPSC C2 and i-mPSC C3 cells (of these, 872 proteins had more than one peptide). A list of all proteins is provided in Supplementary Materials Table S4. 

## 4. Discussion

Pancreatic ductal adenocarcinoma has the worst prognosis of all solid cancers [1], which is mainly due to the late diagnosis of the disease and pronounced resistance to most conventional chemotherapeutic agents [4]. The tumor stroma, which is exceedingly prominent in PDAC, has been attributed an increasingly important role in chemoresistance, although the exact underlying mechanisms are not fully known [34]. So far, stroma-targeting therapies have shown mixed results in clinical studies [12,35,36], which highlights the need for preclinical research into the complex interactions between the cancer cell population and tumor stroma, in which PSC play a central role [37,38]. Currently, much of the research in this field is based on in vitro studies with PSC cultures that may be primary or immortalized and may be of varying origin in terms of species (human or murine), status of the pancreas (normal, chronic pancreatitis or PDAC), and donor age [17,39]. While preliminary data point at differences in transcriptional fingerprints between PSCs from chronic pancreatitis and PDAC [22], the impact of the considerable differences in origin on the phenotype and functional traits has not been investigated, leaving unanswered questions about the comparability of data from studies using different PSC cultures. In this study, a panel of primary and immortalized, human and murine PSC cultures was characterized, and findings reveal considerable differences in basic cellular processes and PSC-specific functions as well as in key interactions with PDAC cell lines (summary in Table 1). 

Morphometric assessment revealed a variable but overall considerably larger size of primary human compared to HPaSteC from normal human pancreas and immortalized PSCs. A characteristic stellate or polygonal shape was found in all PSC cultures, except for the cells of HPaSteC that were thin, spindle-shaped and those of immortalized human PSCs that were roundish. The latter PSC culture also lacked the expression of α-SMA, an activation marker of PSCs, which was found being expressed in the remainder of the PSC panel, albeit at low levels in the HPaSteC culture. As anticipated, cytoplasmic lipid droplets that are typically found in quiescent PSCs in normal pancreas, were absent in the primary human PDAC-derived PSC cultures and present in a minority of cells in HPaSteC which expressed α-SMA at low levels. Interestingly, they were also lacking in the α-SMA-negative i-hPSC culture, but were present to a large extent in the i-mPSC cultures, pointing at a certain divergence and heterogeneity in activation status and marker expression. Cell proliferation was high in HPaSteC, which was unexpected considering the origin of this culture from normal pancreas, in which PSCs are predominantly quiescent [32]. While the proportion of senescent cells was similar in HPaSteC and primary hPSCs, the doubling time of the latter was much longer, indicating a true slower growth pace. Proliferation was high in immortalized PSC cultures, which is the reason why the latter are preferably used for in vitro studies. The continued high proliferation in i-hPSC and i-mPSC cultures is also the reason why these are traditionally referred to as “immortalized” PSCs, despite the fact that the method of “immortalization”, i.e., transfection with SV40 Large T Antigen, results in major disruptions that are more akin to transformation and may explain some of the observed major divergences that are discussed below. 

Remarkable and to some extent unanticipated differences were observed also regarding collagen synthesis, both at base line and following TGF-β stimulation. Basal collagen synthesis was unexpectedly high in HPaSteC, considering the fact that PSCs in normal pancreas are assumed not to produce collagen, unless stimulated [37,40]. This more-than-anticipated basal collagen synthesis may be linked to the above-described features of cell activation in the HPaSteC culture. Basal collagen synthesis was also remarkably high in i-mPSCs but not in i-hPSC, despite the fact that the latter was isolated from a pancreas with chronic pancreatitis [18], in which PSCs are the main effectors of pancreatic fibrosis [41]. Interestingly, TGF-β stimulation of collagen synthesis was observed only in primary PSC cultures, suggesting that immortalized human and murine PSCs lack factors that are required for the transduction of TGF-β stimulation. These findings were substantiated by the observation that all primary human PSC cultures expressed relatively high TGF-β RI, which thereby facilitated TGF-β-induced increased phosphorylation of Smad-2, -3, and resulted in increased collagen synthesis. Interestingly, despite low TGF-β RI, i-hPSCs displayed TGF-β-induced increased phosphorylation of Smad-2, -3; however, this did not translate into increased collagen synthesis, suggesting that other mechanisms may be involved, including the different origin of cells. Failure of TGF-β to induce collagen synthesis in immortalized mouse PSC cultures could be linked to the observed significantly lower expression of TGF-β RI in these cultures. Of particular interest are the complex interactions between PSCs and PDAC, especially the ability of PSCs to influence cancer progression and treatment resistance [41,42,43,44]. Increased DNA synthesis and migration of the cancer cells as well as increased resistance to gemcitabine are possible mechanisms underlying these tumor-promoting effects. As anticipated, and in accordance with the literature [31,43,45], these effects were observed with the conditioned medium from primary human PSC cultures. Interestingly, however, they were lacking when the same cancer cell lines were exposed to conditioned medium from immortalized human and murine PSC cultures. This suggests that secreted factors involved in these interactions are either lacking or present at too low a level to effectuate these crucial alterations in the PDAC cell lines. 

In view of these findings, the composition of the secretome from the various PSC cultures was investigated by proteomics-based analysis. This revealed that, compared to hPSCs, 69% and 43% of the overall 1013 identified proteins were differentially secreted by HPaSteC and i-hPSC, respectively. While these differentially expressed proteins are involved in various cell biological processes, many of these relate to the modeling of the ECM. Interestingly, collagens were found at a considerably lower level in the secretome of immortalized than primary human PSCs.

Taken together, the results of this study confirm that hPSCs have a phenotype that is deemed characteristic of PSCs in PDAC and consists of an activated cell status, low proliferation, TGF-β inducible collagen synthesis, and a profound impact on cancer cells in terms of increased DNA synthesis, cell migration, and resistance to gemcitabine. As anticipated, the proliferation rate was found to be high in i-hPSC, a feature that makes this PSC culture attractive for in vitro studies. However, i-hPSC lacked functions that are considered key to PSCs: increased collagen synthesis following TGF-β stimulation and induction of DNA synthesis, cell migration and resistance to gemcitabine in PDAC cell lines. Similar observations were made for i-mPSC, except for upregulated DNA synthesis in MIA PaCa by clone 2 (i-mPSC C2). HPaSteC cultures surpassed hPSCs in all aspects, except for the expression of α-SMA, which was low. These findings are difficult to reconcile with the origin of this culture from PSCs in normal pancreas and the low expression of α-SMA, which suggests a low-level activation status. While investigations into the reasons for this unexpected phenotype are beyond the scope of this study, isolation by a possibly different method (e.g., density gradient centrifugation) and the recommended use of a culture medium with stellate cell growth supplement may be possible contributions, although the latter had in our experience no significant effect on cell viability when compared with DMEM growth medium that was used for other cultures.

While the identification of the reasons for the observed differences between the various PSC cultures is well beyond the scope of this study, the profound impact of immortalization along with the risk of phenotypic drift as passage numbers increase are likely important causes for the observed differences between primary and immortalized PSC cultures [46]. The status of PSC activation at the time of isolation may also play a role as well as the supplementation of growth-stimulating factors to the culture medium, as it is the case for HPaSteC. Furthermore, it has to be considered that PSC cultures may be heterogeneous and contain subpopulations of PSCs that differ in, for example, activation status, as evidenced by the partial loss of lipid droplets in i-mPSC. In this respect, quantitative—but not qualitative—differences between the three hPSCs point at a degree of functional inter-tumor heterogeneity, also for these non-neoplastic cell populations [47]. Finally, it needs to be mentioned that the hPSCs analyzed in this study were isolated by the outgrowth method, and therefore in culture for several weeks, which may have altered, at least to some degree, their character compared to that of true primary PSCs. 

Interpretation of the results of this study has certain limitations, because the various PSC cultures that are compared differ in origin (i.e., species, age), condition of the donor pancreas (i.e., normal, pancreatitis, PDAC), and whether the cultures were primary or immortalized. These multiple concomitant divergent features impede direct comparison and in particular, they preclude identification of the impact of immortalization on the PSC phenotype. Investigation of the latter requires separate studies in which primary and immortalized human PSCs of the same donor are compared for their phenotypic and genotypic features.

In conclusion, this study reveals significant differences in key phenotypic and functional features between various PSC cultures that are commonly used for in vitro experiments. Awareness of these differences is important when selecting the most appropriate PSC culture for a given experiment as well as when comparing results from studies that used different PSC cultures. Consideration of these differences may explain seemingly conflicting results and facilitate the translation of in vitro findings to clinical application.

## Figures and Tables

**Table 1:** Characteristics of the various pancreatic stellate cell (PSC) cultures.

Cell Culture	hPSCs	HPaSteC	i-hPSC (RLT-PSC)	i-mPSC (C2, C3)
Origin *- Donor species* *- Donor age* *- Pancreas disease status*	Human Adult PDAC	Human Fetal Normal	Human Adult Chronic pancreatitis	Mice Adult Normal
Method of isolation	Outgrowth method	Not known (due to proprietary reasons)	Outgrowth method	Accudenz gradient centrifugation
Immortalization method	No immortalization	No immortalization	Transfection with retrovirus containing SV40 large T antigen and hTERT	Transfection with retrovirus containing SV40 large T antigen
Morphology	Large, polygonal	Long, thin, spindle-shaped	Small, roundish	Small, stellate-shaped
α-SMA expression	Positive (100%)	Positive (100%)	Negative	Positive (72%, 75%)
Vimentin expression	Positive (100%)	Positive (100%)	Positive (100%)	Positive (100%)
Lipid droplets (BODIPY)	Negative	Positive (20%)	Negative	Positive (59%, 63%)
Doubling time (h)	54.9 ± 3.4	25.8 ± 1.4	28.6 ± 2.5	22.0 ± 0.2
TGF-β stimulated collagen synthesis	Upregulated	Upregulated	No change	No change
**Effect of PSC conditioned medium on pancreatic cancer cells (BxPC-3 and MIA PaCa-2):**				
DNA synthesis	Upregulated	Upregulated	No change	No change except: Upregulated for C2 in MIA PaCa-2
Migration	Upregulated	Upregulated	No change	Upregulated
Induction of resistance to gemcitabine	Yes	Yes	No	No

## Supplementary Materials

Supplementary materials are available online.
