# Supplementary material for: Commonly Used Pancreatic Stellate Cell Cultures Differ Phenotypically and in Their Interactions with Pancreatic Cancer Cells
Source: Cells. 2019 Jan 5;8(1):23. doi: 10.3390/cells8010023 (PMC6356867; doi:10.3390/cells8010023)
Supplement: Supplementary file 1 [file cells-08-00023-s001.zip › Supplementary/Supplementary Figure 2.pdf]

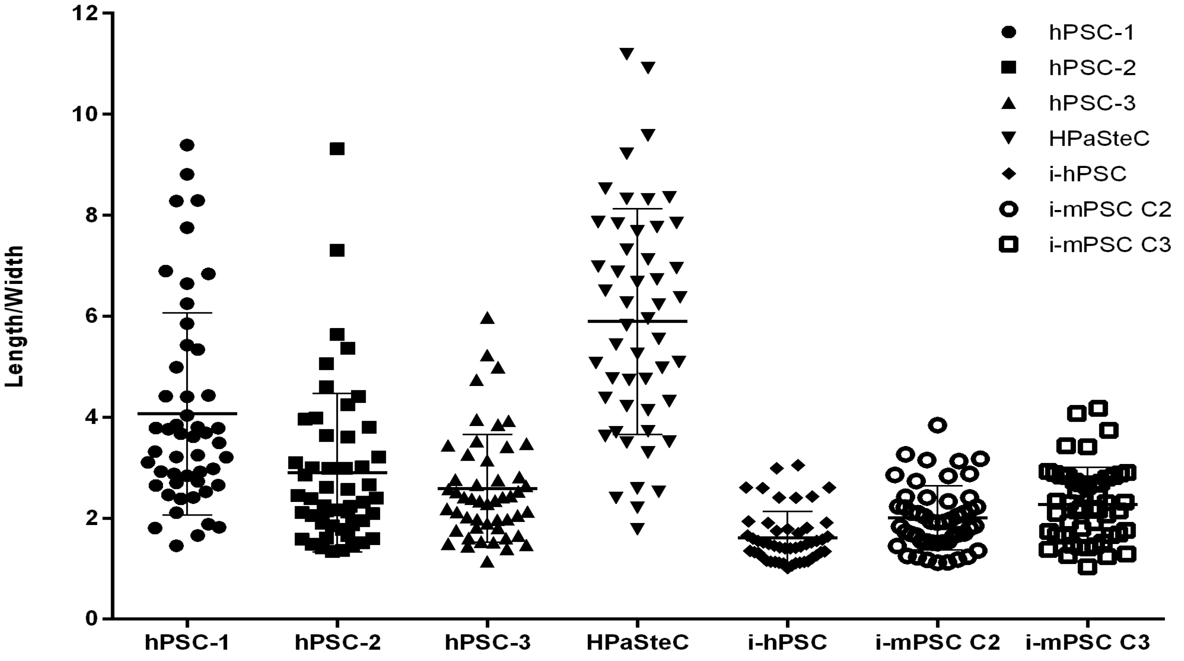

Supplementary Figure 2. Phenotypic characterization of PSCs. Cell size measurement of the various PSC cultures calculated by the length and width ratio of 50 cells for each PSC culture using FIJI software. PSC, pancreatic stellate cell; h-PSC, human primary PDAC-derived PSC culture; HPaSteC, PSCs from normal human pancreas; i-hPSC, immortalized human PSCs; i-mPSC C2 and C3, immortalized mouse PSCs clone 2 and 3.
