# Supplementary material for: Commonly Used Pancreatic Stellate Cell Cultures Differ Phenotypically and in Their Interactions with Pancreatic Cancer Cells
Source: Cells. 2019 Jan 5;8(1):23. doi: 10.3390/cells8010023 (PMC6356867; doi:10.3390/cells8010023)
Supplement: Supplementary file 1 [file cells-08-00023-s001.zip › Supplementary/Supplementary Figure 3.pdf]

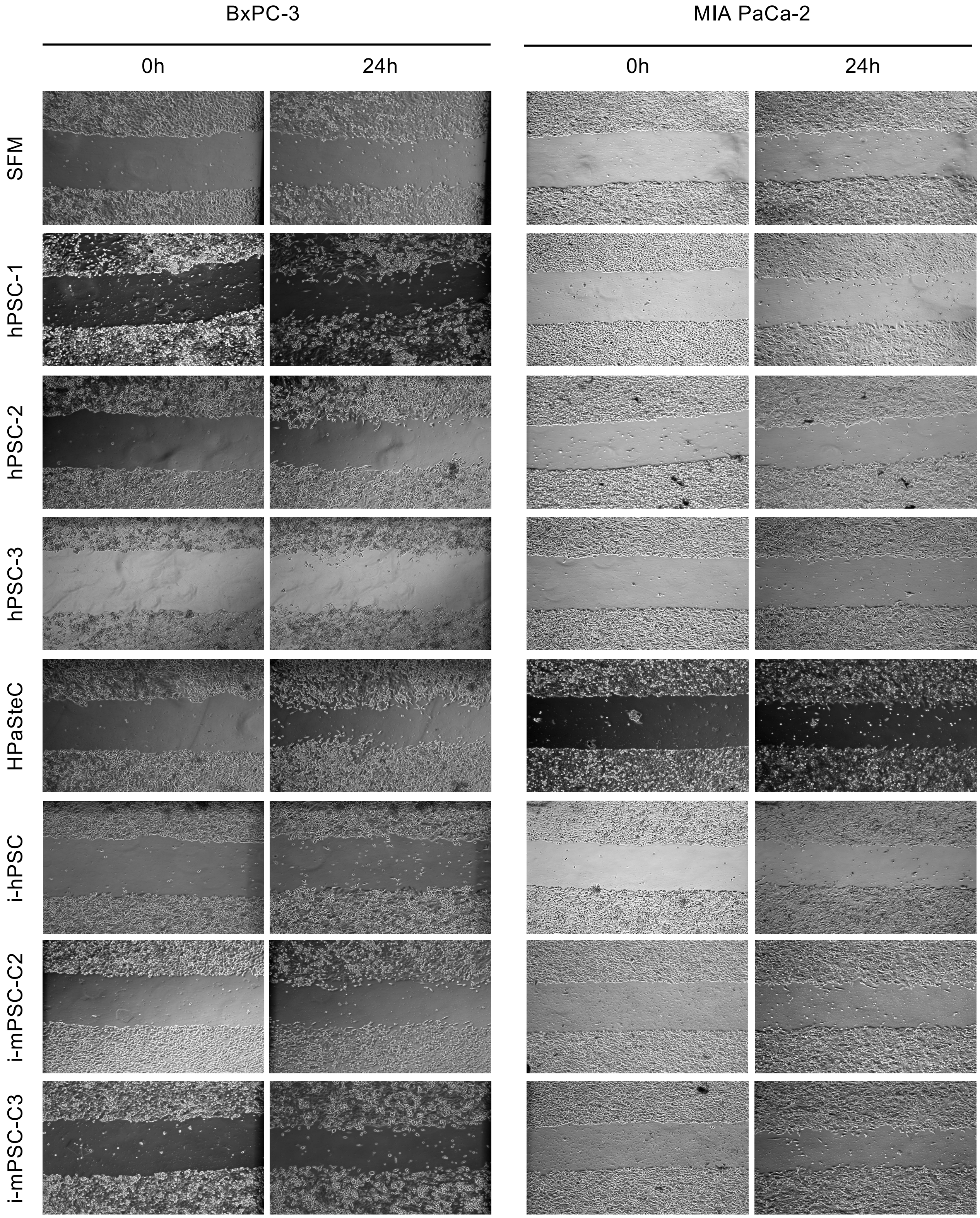

Supplementary Figure 3. Effect of PSC-CM on cancer cell migration. BxPC-3 and MIA PaCa-2 cells were cultured to confluence and scratch wounds were established. The images of the wound area were taken immediately after the scratches and 24 h after incubation with SFM or respective PSC-CM. PSC, pancreatic stellate cell; h-PSC, human primary PDAC-derived PSC culture; HPaSteC, PSCs from normal human pancreas; i-hPSC, immortalized human PSCs; i-mPSC C2 and C3, immortalized mouse PSCs clone 2 and 3; PSC-CM, PSC conditioned medium; SFM, serum-free DMEM.
