# Supplementary material for: Commonly Used Pancreatic Stellate Cell Cultures Differ Phenotypically and in Their Interactions with Pancreatic Cancer Cells
Source: Cells. 2019 Jan 5;8(1):23. doi: 10.3390/cells8010023 (PMC6356867; doi:10.3390/cells8010023)
Supplement: Supplementary file 1 [file cells-08-00023-s001.zip › Supplementary/Supplementary Figure 1.pdf]

A

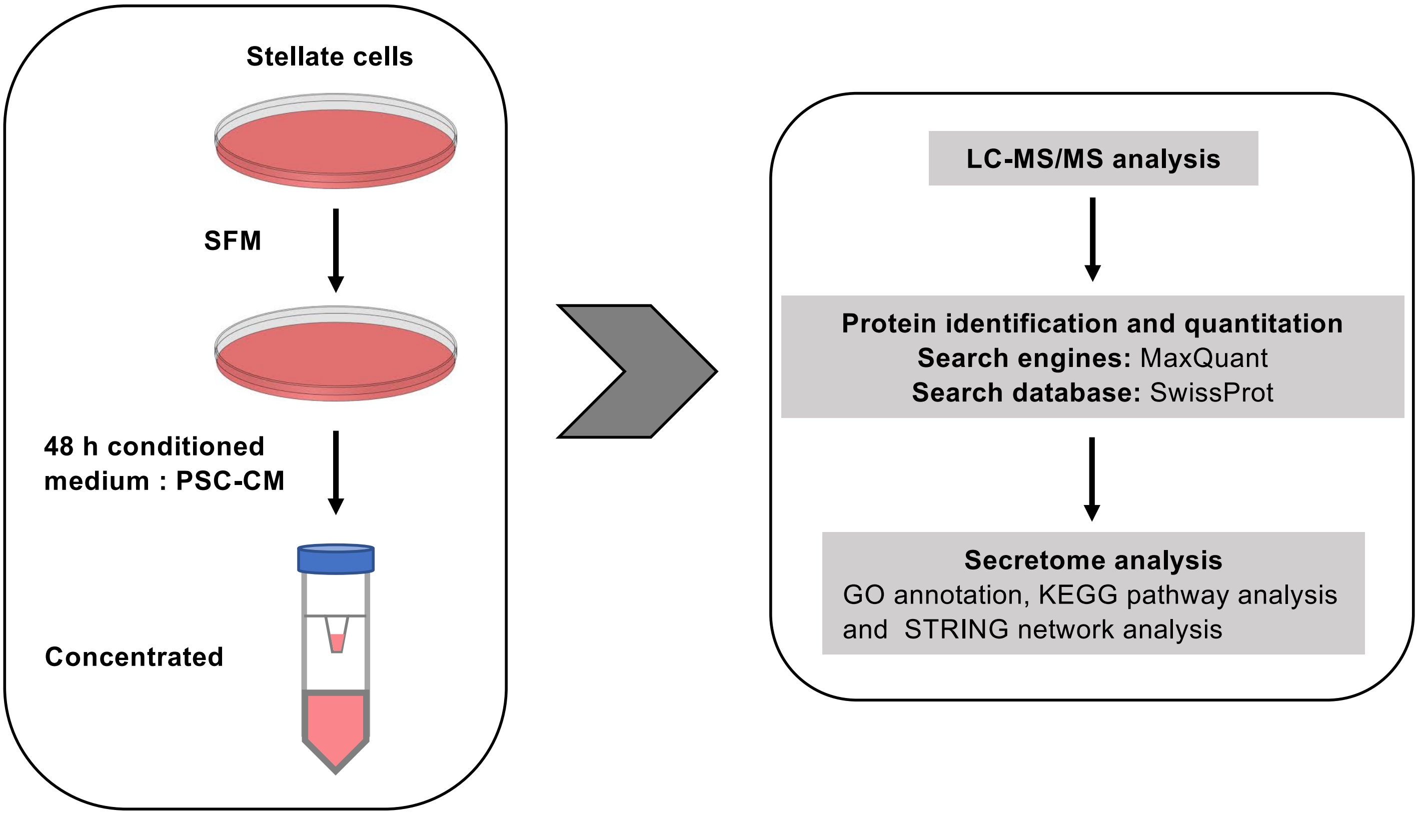

B

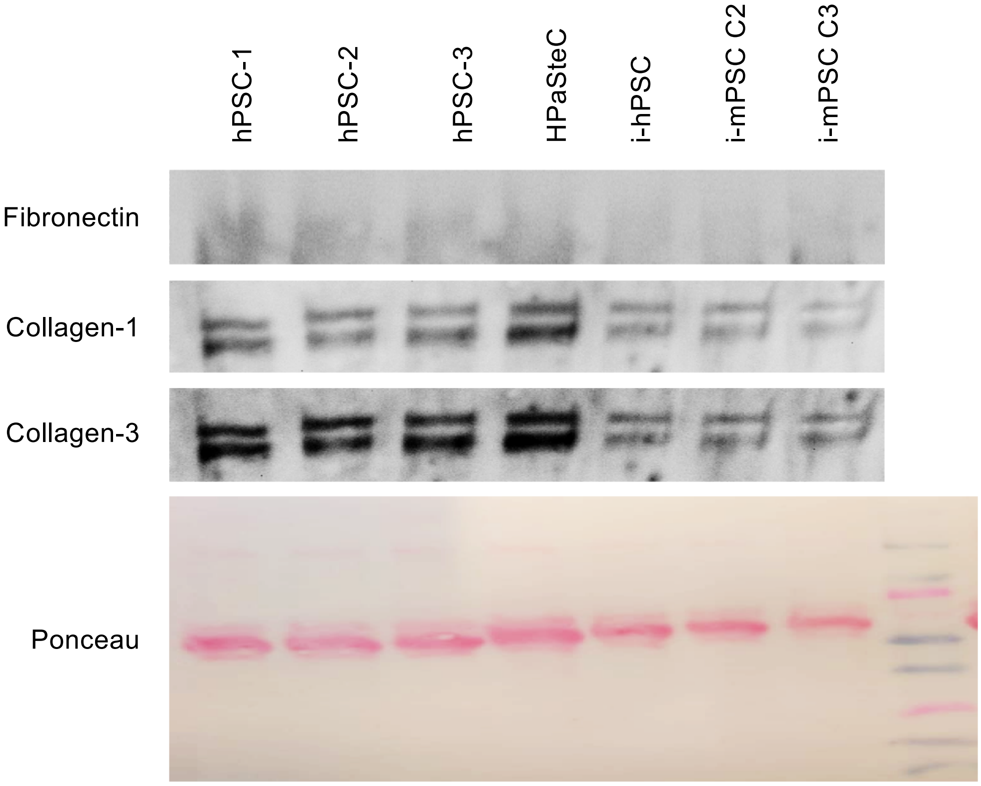

Supplementary Figure 1. Secretome analysis and protein identification. (A) Flow-chart describing procedure for conditioned medium collection, processing and analysis for proteome of secretome. PSC-CM, pancreatic stellate cell conditioned medium, SFM, serum-free DMEM. (B) The conditioned media samples (~5 ml) were concentrated down to 250 μl using 10 kDa cut off Amicon Ultra centrifugal filter devices. Further the proteins were evaluated for immunoblotting using antibodies from Abcam (Cambridge, UK): anti-Fibronectin (ab2413), anti-Collagen 1 (ab34710), anti-Collagen 3 (ab7778). Ponceau staining is shown for loading control.
